# Intestinal flora induces depression by mediating the dysregulation of cerebral cortex gene expression and regulating the metabolism of stroke patients

**DOI:** 10.3389/fmolb.2022.865788

**Published:** 2022-11-30

**Authors:** Xuebin Li, Guangshun Han, Jingjie Zhao, Xiaohua Huang, Yun Feng, Junfang Huang, Xuequn Lan, Xiaorui Huang, Zechen Wang, Jiajia Shen, Siyuan He, Qiuhao Li, Jian Song, Jie Wang, Lingzhang Meng

**Affiliations:** ^1^ Center for Systemic Inflammation Research (CSIR), School of Preclinical Medicine, Youjiang Medical University for Nationalities, Baise, Guangxi, China; ^2^ Department of Neurology, The Affiliated Hospital of Youjiang Medical University for Nationalities, Baise, Guangxi, China; ^3^ Department of Neurology, Liuzhou People’s Hospital, Liuzhou, Guangxi, China; ^4^ Life Science and Clinical Research Center, The Affiliated Hospital of Youjiang Medical University for Nationalities, Baise, Guangxi, China; ^5^ Institute of Cardiovascular Sciences, Guangxi Academy of Medical Sciences, Nanning, Guangxi, China; ^6^ Department of Renal Diseases, The Affiliated Hospital of Youjiang Medical University for Nationalities, Baise, Guangxi, China

**Keywords:** intestinal flora, depression, cerebral cortex, metabolism, stroke

## Abstract

Post-stroke depression (PSD) is a common cerebrovascular complication characterized by complex pathogenesis and poor treatment effects. Here, we tested the influence of differentially expressed genes (DEGs), non-targeted metabolites, and intestinal microbes on the occurrence and development of PSD. We acquired gene expression profiles for stroke patients, depression patients, and healthy controls from the Gene Expression Omnibus database. After screening for DEGs using differential expression analysis, we identified common DEGs in stroke and depression patients that were considered to form the molecular basis of PSD. Functional enrichment analysis of DEGs also revealed that the majority of biological functions were closely related to metabolism, immunity, the nervous system, and microorganisms, and we also collected blood and stool samples from healthy controls, stroke patients, and PSD patients and performed 16S rDNA sequencing and untargeted metabolomics. After evaluating the quality of the sequencing data, we compared the diversity of the metabolites and intestinal flora within and between groups. Metabolic pathway enrichment analysis was used to identify metabolic pathways that were significantly involved in stroke and PSD, and a global metabolic network was constructed to explore the pathogenesis of PSD. Additionally, we constructed a global regulatory network based on 16S rDNA sequencing, non-targeted metabolomics, and transcriptomics to explore the pathogenesis of PSD through correlation analysis. Our results suggest that intestinal flora associates the dysregulation of cerebral cortex gene expression and could potentially promote the occurrence of depression by affecting the metabolism of stroke patients. Our findings may be helpful in identifying new targets for the prevention and treatment of PSD.

## 1 Introduction

Stroke is one of the major causes of death in China and is characterized by high morbidity, disability, recurrence, and mortality (Boursin et al., 2018). Stroke is classified into two types, including ischemic and hemorrhagic stroke, that occur as a result of reduced blood flow to the brain that ultimately causes cell and tissue necrosis. Post-stroke depression (PSD) is a common complication of stroke and has been reported in 20–50% of stroke patients worldwide (Das and Rajanikant, 2018). Although the pathogenesis of PSD remains unclear, it has been associated with monoamine neurotransmitters, immune imbalance, and neurogenetic pathways (Fang and Cheng, 2009). PSD is considered to be a consequence of multiple interactions among biological, psychosocial, and multifactorial factors, and based on this, deepening our understanding of how PSD occurs is critical for treatment and prevention.

Previous studies have demonstrated that disordered intestinal flora can cause a range of diseases (Pascale et al., 2018; Fan and Pedersen, 2021), including neurological diseases. These microbiota regulate the brain-gut axis *via* neural-, endocrine-, metabolism-, and immunity-related mechanisms to modulate brain function (Junges et al., 2018). Changes in intestinal microbiota have been reported to play a role in the occurrence and development of neurological diseases such as dementia, Alzheimer’s Disease (Alkasir et al., 2017; Tsunoda, 2017), autism (Strati et al., 2017), and depression (Slyepchenko et al., 2017). An imbalance in intestinal flora can also affect the outcome of stroke *via* changes in bacterial populations and their translocation and alterations in intestinal cell metabolites and immune regulation (Wen and Wong, 2017). Furthermore, gut microbes can induce depression by causing inflammatory responses and imbalances in monoamine neurotransmitters and neurotrophic factors and by activating the hypothalamic-pituitary-adrenal axis (Dinan and Cryan, 2019; Links between gut microbes and, 2019). However, there is no clear understanding of the exact mechanism by which intestinal flora can influence the occurrence and development of PSD.

Another factor that increases susceptibility to diseases is the existence of metabolic abnormalities. Systemic diseases affecting the brain are often associated with severe metabolic disorders (Heindel et al., 2017). When the blood-brain barrier is compromised, the brain tissue is affected by the internal biochemical environment, thus resulting in metabolic changes that in turn lead to brain dysfunction (Gonzalez et al., 2016). Previous studies have demonstrated that there is a significant association between abnormalities in the metabolism of glucose and lipids and the occurrence and prognosis of stroke (Zhai et al., 2006) and that changes in lipid and iron metabolism are significantly associated with the occurrence of depressive events (Oliveira et al., 2017).

This study aimed to gain a better understanding of the pathogenesis of PSD by examining the role played by intestinal flora, metabolites, and differentially expressed genes (DEGs) in the occurrence and development of PSD. These findings can help guide the clinical treatment of PSD.

## 2 Materials and methods

### 2.1 Sample collection

We collected blood and stool samples from all patients who were admitted to Youjiang Medical University for Nationalities between August 2019 and November 2020. Patients with hemiplegia, speech disorder, hemianesthesia, distortion of commissure and stroke history, with CT/MRI evidence (while the other neuroinflammation diseases such as encephalitis, tumor *etc.* Were excluded) were diagnosed and recruited into stroke group; stroke patients with depression, frustration and/or interest loss persistent over 1 week were diagnosed and recruited into PSD group. The HDRS score was employed to evaluate the severity of depression, to minimize the system error, only those patients scored between 7 and 17 (mild depression) was recruited. Patients ranged in age from 44 to 73 years (no significant difference was found in the HDRS score between PSD patents <59 years old and ≥59 years old, data not shown). This study was approved by the ethics committee of Youjiang Medical University for Nationalities. Written informed consent was obtained from the patients to allow for their anonymized data to be collected and analyzed for research purposes. Prior to conducting further genetic analyses, patients were stratified into three groups that included healthy controls (n = 30, group A), stroke patients (n = 34, group B), and PSD patients (n = 26, group C).

### 2.2 16S rDNA

#### 2.2.1 16S rDNA sequencing

##### 2.2.1.1 Extraction of genome DNA

The total genomic DNA from the samples was extracted using the CTAB/SDS method. DNA concentration and purity were monitored using a 1% agarose gel. DNA was diluted to 1 ng/μl using sterile water at volumes that were dependent upon the initial DNA concentration.

##### 2.2.1.2 Amplicon generation

Primers: 16S V3-V4: 341F-806R; 18S V9: 1380F-1510R, ITS1: ITS1F- ITS2R.

Specific primers possessing barcodes were used to amplify the 16S/18S rRNA gene. The PCR reactions were performed using 30 µl reactions with 15 µl of Phusion^®^ High Fidelity PCR Master Mix (New England Biolabs) and 0.2 µl of forward and reverse primers.

##### 2.2.1.3 PCR products quantification and qualification

An equal volume of 1X loading buffer (containing SYB green) was mixed with the PCR products and then electrophoresed on a 2% agarose gel for detection. Samples exhibiting bright main bars between 400 and 450 bp were selected for further experiments.

##### 2.2.1.4 Mixing and purification of PCR products

The PCR products were mixed at equal density ratios. Then, an AxyPrepDNA Gel Extraction Kit (AXYGEN) was used to purify the mixed PCR products.

##### 2.2.1.5 Library preparation and sequencing

Sequencing libraries were generated using the NEB Next®Ultra™ DNA Library Prep Kit from Illumina (NEB, USA), and indexing codes were added according to the manufacturer’s instructions. A Qubit™ 2.0 Fluorometer (Thermo Scientific) and an Agilent Bioanalyzer 2,100 system were used to assess the library quality. Finally, using the Illumina Miseq/HiSeq2500 platform, the libraries were sequenced, and 250bp/300bp paired-end reads were generated.

#### 2.2.2 16S rDNA data analysis

##### 2.2.2.1 Paired-end reads assemblies

The paired-end reads from the original DNA fragments were merged using FLASH that was designed to combine fragments into pairs when at least part of the readings overlapped with those generated at the other end of the same DNA fragment. Paired reads were assigned to each sample based on a unique barcode.

### 2.3 OTU cluster and species annotation

The UPARSE-OTU and UPARSE-OTUref algorithms of the UPARSE package were used for the sequence analysis. The alpha (within-sample) and beta (between-sample) diversities were analyzed using internal Perl scripts in which sequences with ≥97% similarity were assigned to the same OTU. We selected a representative sequence for each OTU and annotated the taxonomic information for each representative sequence using the RDP classifier. To calculate alpha diversity, we sparsed the OTU table and computed three metrics that included Chao1 that estimates species abundance, Observed Species that estimates the number of unique OTUs for each sample, and the Shannon index. The three metrics provided the basis for the rarity curves. For quality control, we observed the sequencing results with cluster density of 76 ± 8.5 K/mm2, and only those >85% of the clusters were used for down-streaming analysis, besides, a quality score of Q > 31 was assigned to 94.5% of all bases from both reads. Only the reads passed filtering were used for down-streaming analysis.

### 2.4 Phylogenics distance and community distribution

The relative abundance of bacterial diversity was displayed using a Krona plot. Prior to cluster analysis, principal component analysis (PCA) was utilized to reduce the dimensionality of the original variables using the QIIME package. We used unweighted UniFrac distances for principal coordinate analysis (PCoA) and unweighted arithmetic mean pair group method (UPGMA) clustering. The principal coordinates from complex multidimensional data were visualized using PCoA. UPGMA clustering is a hierarchical clustering method that can be used to interpret the distance matrix.

### 2.5 Statistical analysis

The 16S rDNA analysis was performed to identify and quantify microbes in the blood samples collected from the patients (Supplementary Table S1). We used the Statistical Analysis of Metagenomic Profiles software to examine differences across the three groups in regard to the abundances of individual microbial taxa found in the samples. A quantitative analysis of biomarkers within each group was conducted based on linear discriminant analysis effect size (LEfSe). This method was designed to analyze datasets where the number of species is much higher than is the number of samples, and this method can be used to determine the features that are most likely to explain the differences between biological classes by testing for statistical significance and biological consistency and also by estimating the effect sizes of predicted biomarkers.

### 2.6 Untargeted metabolomics

#### 2.6.1 Sample collection and preparation

Fasting blood samples were collected in 5 ml Vacutainer tubes containing the chelating agent ethylene diamine tetraacetic acid (EDTA), and the samples were centrifuged for 15 min (1,500 g at 4°C). Each aliquot (150 μl) of the plasma sample was stored at -80°C until UPLC-Q-TOF/MS analysis. The plasma samples were thawed at 4°C, and 100 μl aliquots were mixed with 400 μl of cold methanol/acetonitrile (1:1, v/v) to remove the protein. The mixture was centrifuged for 15 min (14,000 g at 4°C). The supernatant was then dried using a vacuum centrifuge. For LC-MS analysis, the samples were re-dissolved into 100 μl of acetonitrile/water (1:1, v/v) solvent.

#### 2.6.2 LC-MS/MS analysis

The analysis was performed using an ultra-high-performance liquid chromatograph (1290 Infinity LC, Agilent Technologies) combined with a quadrupole time-of-flight instrument (AB Sciex TripleTOF 6,600) from Shanghai Applied Protein Technology Co., Ltd. Samples were analyzed using a 2.1 mm × 100 mm ACQUIY UPLC BEH 1.7 µm column (Waters, Ireland) for HILIC separation. In the ESI positive and negative modes, the mobile phase consisted of A = 25 mM ammonium acetate and 25 mM ammonium hydroxide aqueous solution and B = acetonitrile. The gradient was 85% B at 1 min, linearly decreased to 65% at 11 min, subsequently decreased to 40% at 6s, and then held for 4 min. It was then increased to 85% at 6s using a 5 min re-equilibration period.

A 2.1 mm × 100 mm ACQUIY UPLC HSS T3 1.8 µm column (Waters, Ireland) was used for RPLC separation. In ESI positive mode, the mobile phase was A = water plus 0.1% formic acid and B = acetonitrile plus 0.1% formic acid, and in ESI negative mode, the mobile phase was A = 0.5 mM ammonium fluoride in water and B = acetonitrile. The gradient was 1% B for 1.5 min and then increased linearly to 99% in 11.5 min and held for 3.5 min. It was then decreased to 1% over a 6s time period, and a re-equilibration period of 3.4 min was subsequently applied. The flow rate of the gradient was 0.3 ml/min, and the column temperature was maintained at 25°C. Each sample was injected with a 2 µl aliquot.

The instrument of MS-only acquisition was set to acquire within the m/z range of 60–1000 Da, and for the TOF MS scan, the accumulation time was set to 0.20 s/spectrum. The instrument for automated MS/MS acquisition was set to acquire within the m/z range of 25–1000 Da, and the accumulation time for the product ion scan was set to 0.05 s/spectrum. The product ion scan was acquired using information-dependent acquisition (IDA), and the high-sensitivity mode was selected for acquisition. The parameters were set as follows: the collision energy (CE) was fixed at 35 V with ±15 eV; declustering potential (DP) was 60 V (+) and −60 V (−); excluding isotopes within 4 Da; candidate ions to monitor per cycle set to 10.

#### 2.6.3 Data processing

Prior to import into the XCMS software, the raw mass spectrometry data (wiff.scan files) were converted to MzXML files using ProteoWizard MSConvert. For peak extraction, the following parameters were used: centWave m/z = 25 ppm, peak width = c (10, 60), and prefilter = c (10, 100). For peak grouping, bw = 5, mzwid = 0.025, and minfrac = 0.5. The annotation of isotopes and adducts was completed using the Collection of Algorithms of MEtabolite pRofile Annotation (CAMERA). Among the extracted ion signatures, variables exhibiting greater than 50% of non-zero measurements in at least one group were retained. Metabolite identification was performed by comparing accurate m/z values (<25 ppm) and MS/MS spectra with available authentic standards established in an in-house database.

#### 2.6.4 Statistical analysis

Untargeted metabolomics was conducted using stool samples collected from the patients (Supplementary Table S2). After normalization to total peak intensity, we used the ‘*muma*’ package in R to perform univariate statistical analyses to identify differential metabolites. The metabolites identified were considered to be differentially expressed if log_2_ (fold change [FC]) > 1.5 or log_2_(FC) < 0.67 and *p* < 0.05. Metabolic pathway enrichment analysis was performed using MetaboAnalyst (https://www.metaboanalyst.ca/) to explore the biological functions associated with the differential metabolites.

### 2.7 Transcriptomics

#### 2.7.1 Data quality and preprocessing of data from stroke patients and controls

The GSE56267 dataset (Huttner et al., 2014) that is based on the GPL11154 platform includes data from seven cortical ischemic stroke tissues and six control cortex samples. This dataset was used to screen for DEGs in stroke patients compared to gene levels in controls. Paired-end RNA-seq data (SRP040622) were acquired from the National Center for Biotechnology Information-Sequence Read Archive (NCBI-SRA; http://www.ncbi.nlm.nih.gov/sra), and the data were collected using the ‘prefetch’ function in the SRA Toolkit (https://www.ncbi.nlm.nih.gov/sra/docs/toolkitsoft/) and saved as 13 files (SRR1206035, SRR1206036, SRR1206037, SRR1206038, SRR1206039, SRR1206040, SRR1206041, SRR1206042, SRR1206043, SRR1206044, SRR1206045, SRR1206046, and SRR1206047). The 13 paired SRA files (two groups) were converted into fastq files (13 files) using the ‘fastq-dump’ and’ split-files’ functions.

At this stage, the quality of all datasets was assessed before and after trimming the adaptors and initiating the preprocessing steps using the FastQC tool (https://www.bioinformatics.babraham.ac.uk/projects/fastqc/) (Saddala et al., 2019). Finally, we removed any low-quality reads by trimming the bases from the 3′ and 5’ ends and maintaining a Phred score ≤30 according to the Trimmomatic-0.36 tool (Léránth and Hámori, 1970). After cleaning and trimming the low-quality reads and removing the adaptors, we were able to retain more than 96% of good-quality reads in each stage. The cleaned reads were used for transcriptome assembly analysis.

#### 2.7.2 Reference-based assembly of stroke patients and controls

All datasets were assembled separately with reference to the *Homo sapiens* genome using Bowtie 1.2.2 (Langmead et al., 2009). First, this software created an index of genome files and aligned short reads to the reference genome. Then, RNA-seq analysis based on Expectation-Maximization (Wu et al., 2011) was used to estimate the number of RNA-seq fragments that mapped to each contig with gene annotations in a gene transfer format file. As the abundance of individual transcripts can vary greatly between samples, reads from each sample were examined individually to derive sample-specific abundance values.

#### 2.7.3 Processing of data from patients and controls

The expression profile data from depression patients and control samples in our study were derived from a published dataset (Hagenauer et al., 2018) available in the Gene Expression Omnibus database (GEO: GSE92538). The GSE92538 dataset that is based on the GPL10526 platform contains data from 29 patients with depression and 56 control samples. The source of the sample tissue in this dataset was the dorsolateral prefrontal cortex. The ‘justRMA’ methods in the ‘*affy*’ package (Gautier et al., 2004) in R were applied to normalize the raw data. If one gene corresponded to multiple probes, the average expression value of these probes was considered to be the expression value of the gene.

### 2.8 DEGs and enrichment analysis

The ‘*limma*’ package (Yu et al., 2019) in R was used to analyze DEGs between the case (atheroma plaque) samples and control samples. Statistical significance was set at *p* < 0.05. The ‘*clusterProfiler*’ package (Yu et al., 2012) in R was used to functionally analyze the enriched pathways in which the DEGs were involved based on Gene Ontology (GO) terms and Kyoto Encyclopedia of Genes and Genomes (KEGG) pathways. Statistical significance was set at *p* < 0.05.

Gene set enrichment analysis (GSEA) was performed using GSEA software (Subramanian et al., 2005) with reference to the gene sets c2. cp.kegg.v6.2., symbols. gmt, and c5. bp.v7.0. entrez.gmt, all of which were downloaded from the Molecular Signature Database (http://software.broadinstitute.org/gsea/index.jsp) (Liberzon et al., 2015). Statistical significance was defined as a nominal *p*-value < 0.05 and a false discovery ratio <0.25.

## 3 Results

### 3.1 Common molecular characteristics of stroke and depression are the basis of molecular dysregulation of PSD

**FIGURE 1:**
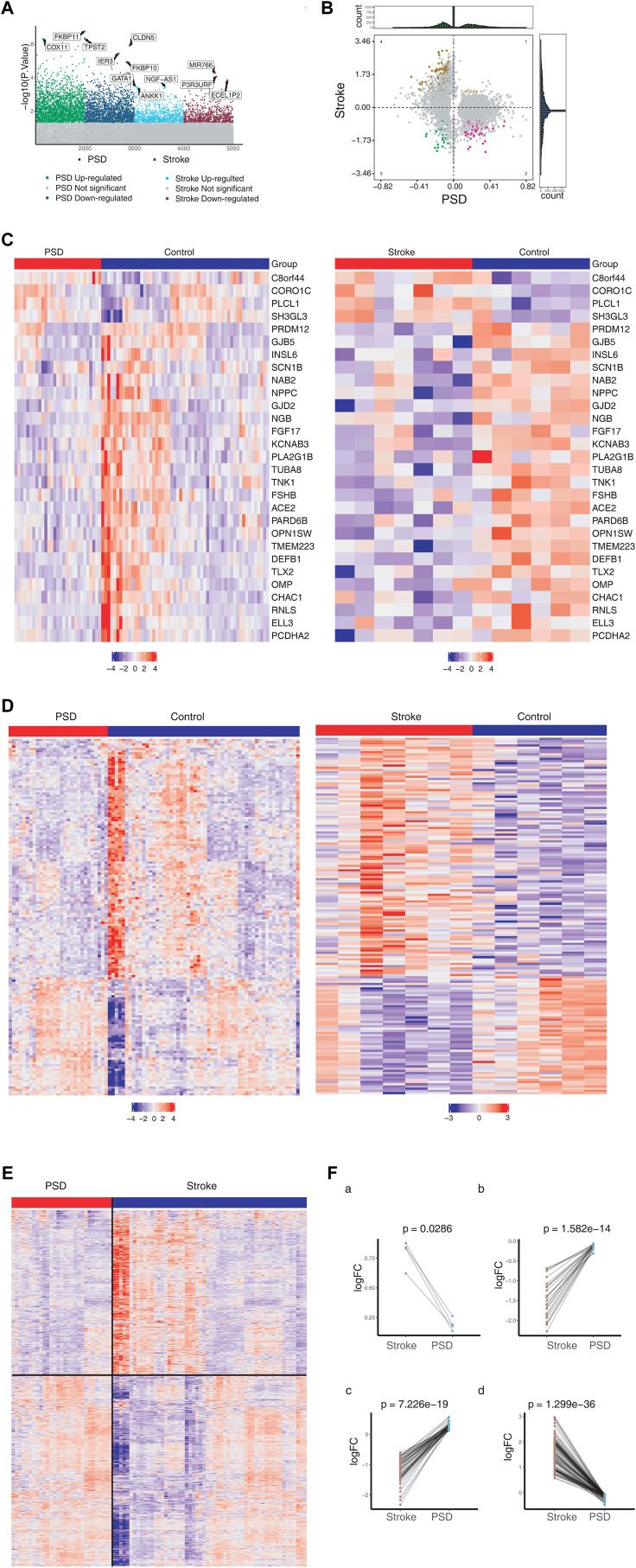
Molecular characteristics of stroke and PSD patients. **(A)**. Manhattan diagram depicting dysregulated genes in stroke and PSD patients. **(B)**. Quadrant chart presenting the expression pattern of genes in stroke and PSD patients. **(C)**. Heatmap indicating consistent upregulation of gene expression in stroke and PSD patients. **(D)**. Heatmap indicating genes that are dysregulated in opposite directions in stroke and PSD patients. **(E)**. Heatmap revealing the expression of depression-specific genes in PSD patients and stroke. **(F)**. Paired scatterplot depicting the expression of genes in stroke and PSD patients.

For the control group, we identified 1,492 DEGs in stroke patients and 4,376 DEGs in patients with depression (Figure 1A). Messenger RNA (mRNA) expression pattern analysis revealed that 29 DEGs were upregulated and 4 DEGs were downregulated in both stroke and PSD patients (Figure 1B). These genes were used to define the molecular signature for PSD. In contrast, the DEGs that were upregulated in stroke patients but downregulated in PSD patients (or *vice versa*) were defined as the molecular signature of recovery from PSD. We observed that the expression pattern of these two types of DEGs exhibited the ability to distinguish stroke or PSD patients from control samples (Figures 1C,D). DEGs that were expressed only in PSD patients (not in stroke patients) were defined as depression-specific genes, and their expression pattern could be used to distinguish PSD patients from healthy controls (Figure 1E).

We identified various gene expression profiles that may contribute to depression. Genes exhibiting |log FC|_st_> 0 and |log2FC|_De_ > 0 maintained high expression in stroke patients but low expression during recovery (Figure 1Fa). Genes with |logFC|_St_ < 0 and |logFC|_De_ < 0 maintained low expression during stroke and recovery (Figure 1Fb). Genes |logFC|_St_ > 0 and |logFC|_De_ < 0 “over-recovered” during stroke recovery based on the observation that their levels during recovery *exceeded* their levels in controls (Figure 1Fc). Genes exhibiting |logFC|_St_ < 0 and |logFC|_De_ > 0 also “over-recovered” during stroke recovery based on the observation that their levels during recovery *fell below* the levels in controls (Figure 1Fd).

### 3.2 Metabolic abnormalities and immune disorders are important drivers of PSD

**FIGURE 2:**
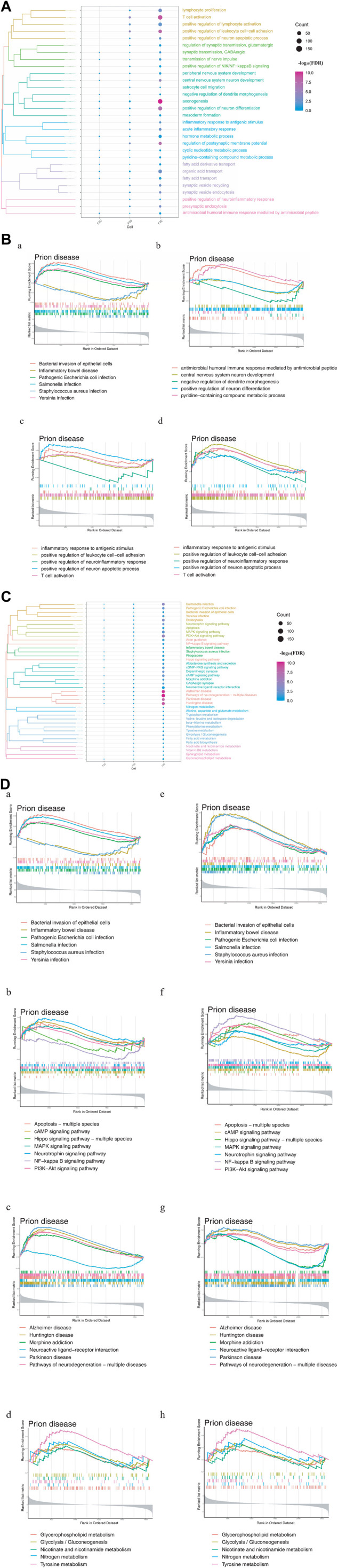
Enrichment analysis of differentially expressed genes (DEGs) in stroke and PSD patients. **(A)**. Biological processes: F2C represents the genes common to stroke and PSD; F2D represents genes that potentially mediate PSD based on excessive changes in expression during stroke recovery; F2E represents depression-specific genes. **(B)**. GSEA. Panels **(a,b)** use common and specific genes associated with stroke and PSD patients as the preset gene set for GSEA. Panels **(c,d)** use depression-specific genes as the preset gene set for GSEA. **(C)**. KEGG pathway: F2C represents the genes common to stroke and PSD; F2D represents genes that potentially mediate depression based on excessive changes in expression during stroke recovery; F2E represents depression-specific genes. **(D)**. GSEA-KEGG pathway: the KEGG pathways were divided into four types according to the classification of intestinal flora, signaling pathways, brain disease, and metabolism (from left to right).

To explore the driving factors that may induce depression after stroke, enrichment analysis was performed on three types of DEGs, including those common to stroke and depression, those whose expression differed substantially between stroke and recovery, and those whose expression was altered only in depression. The GO enrichment results revealed that these three types of DEGs were involved in biological processes (BPs) associated with metabolism, immunity, microbes, and nerves (Figure 2A). Depression-specific genes play a significant role in the activation of T cells and xenogenesis.

GSEA indicated that the BPs associated with the genes common to stroke and depression and also depression-specific genes were significantly associated with immunity and neuronal differentiation. Additionally, BPs associated with depression-specific genes were associated with neuroinflammation, immune inflammation, and apoptosis (Figure 2B). KEGG enrichment analysis revealed that these three types of DEGs were involved in pathways related to intestinal flora, metabolism, brain disease, and immunity (Figure 2C). GSEA also demonstrated that the KEGG pathways related to metabolism, brain disease, immunity, and intestinal flora were enriched in patients with stroke and depression (Figure 2D).

### 3.3 Non-targeted metabolomics can identify key metabolic pathways in blood samples from stroke patients

We observed overlaps in the intensity and retention time of each chromatographic peak in the quality control (QC) samples (Supplementary Figures S1A,B). The correlation coefficients between the QC samples were >0.9, thus indicating good repeatability (Supplementary Figure S1C). More than 80% of the peaks in the QC sample possessed a relative standard deviation of ≤30%, thus indicating the high stability of the instrument analysis system (Supplementary Figure S1D). Based on Hotelling’s T2 test, all QC samples were within the 99% confidence interval, thus indicating that the experiment was reproducible (Supplementary Figure S1E). Fluctuations in the QC samples were within ±3 standard deviations and were reflective of the normal fluctuation of the instrument (Supplementary Figure S1F).

**FIGURE 3:**
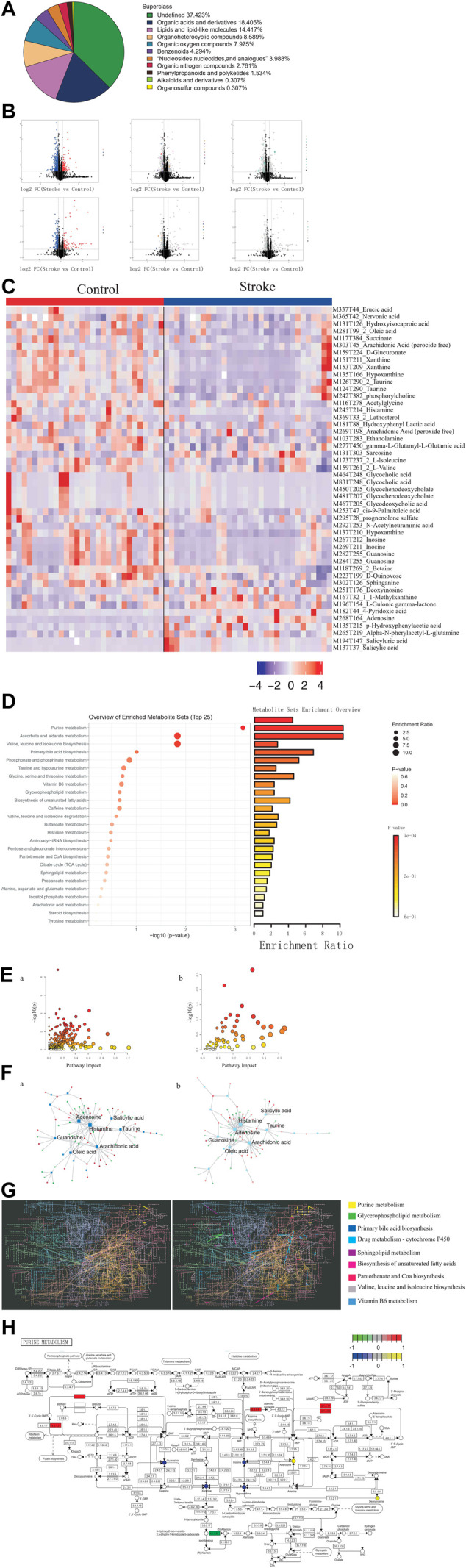
Identification of differential metabolites and dysregulated metabolic pathways in stroke patients. **(A)**. Proportion of the identified metabolites in each chemical classification. **(B)**. Volcano plot depicting differences in metabolites between stroke patients and controls. **(C)**. Heatmap depicting the abundance of differential metabolites in stroke patients and controls. **(D)**. Bubble-bar graph depicting the metabolic pathways associated with different metabolites. **(E)**. KEGG metabolic network diagram. **(F)**. Interaction network of differential metabolites (squares) and differentially expressed genes (circles). **(G)**. KEGG global metabolic network highlighting the pathways possessing significant enrichment scores. **(H)**. Pathway map diagram depicting depression-specific dysregulated genes (in green), genes common to stroke and depression (in red), depression-specific abnormal metabolites (in blue), and abnormal metabolites common to stroke and depression (in yellow).

Among the metabolites with chemical classifications, organic acids and derivatives were found in large concentrations, while organosulfur compounds were found in small concentrations (Figure 3A). Compared to the controls, we identified 3,443 dysregulated metabolites in stroke patients (*p* < 0.05) (Figure 3B). We also determined that these dysregulated metabolites could be used to distinguish between stroke patients and controls (Figure 3C).

Compared to the controls, there were several metabolic pathways that were significantly enriched by differential metabolites in stroke patients, including ascorbate and aldarate metabolism and also valine, leucine, and isoleucine biosynthesis (Figure 3D). Metabolic pathway enrichment analysis was performed based on DEGs and differential metabolites (Figure 3E). Furthermore, we observed extensive interactions between the differential metabolites and DEGs (Figure 3F). The KEGG global metabolic network analysis also revealed that the purine metabolism pathway possessed the most significant enrichment score (Figure 3G) and that DEGs and differential metabolites play an important role in purine metabolism (Figure 3H).

### 3.4 Atlas of abnormal blood metabolism in patients with PSD

**FIGURE 4:**
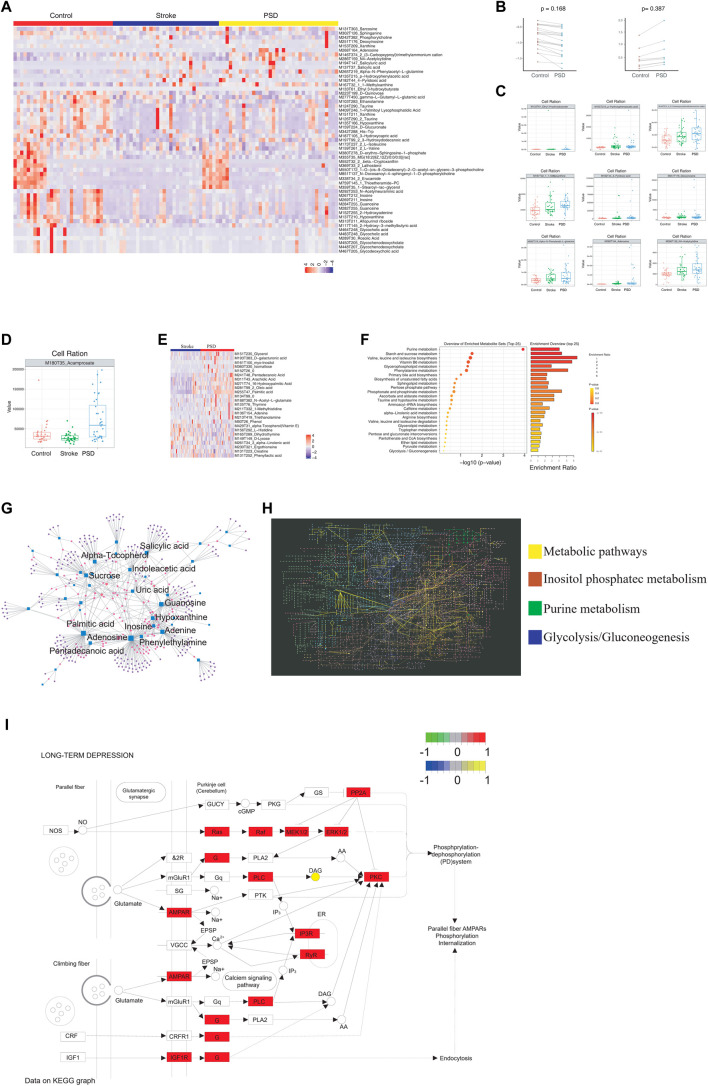
Atlas of abnormal blood metabolism in patients with PSD. **(A)**. Heatmap depicting abnormal metabolites common to stroke and PSD patients. **(B)**. Paired scatterplot depicting continuous changes in total abnormal metabolites. **(C)**. Box plot depicting abnormal metabolites that continue to increase during the process from control-stroke-PSD. **(D)**. Box plot depicting abnormal metabolites associated with excessive recovery during the process from control-stroke-PSD. **(E)**. Heatmap depicting differences in metabolites between stroke and post-stroke depression patients. **(F)**. Metabolic pathway enrichment analysis. **(G)**. Network diagram depicting genes (circles) and metabolites (squares). The size of the dot represents the degree value. **(H)**. KEGG global metabolic network depicting differentially expressed genes and differential metabolites between PSD and stroke patients. Significant pathways are highlighted with a specific color. **(I)**. Pathway map of “long-term depression”.

To identify the metabolic abnormalities in patients with PSD, differential expression analysis was performed using non-targeted metabolomics sequencing data. Patients with PSD exhibited 5,408 differential metabolites when compared to control samples and 1,749 differential metabolites when compared to stroke patients (Supplementary Figure S2A). There were 53 abnormal metabolites common to PSD and stroke patients, and these metabolites could represent the metabolic basis of stroke as a precondition for depression (Figure 4A).

Among the common abnormal metabolites, we observed a decrease in 21 differential metabolites and an increase in seven differential metabolites during the development of stroke into PSD (Figure 4B). We also examined the abundance of these metabolites in all three patient groups (Supplementary Figure S2B and Supplementary Figure S4C). For example, MI80T35_Acamprosate decreased in stroke patients but increased significantly during the process of excessive recovery during the development of PSD (Figure 4D). Furthermore, we determined that these different metabolites in PSD patients can help distinguish between PSD and stroke patients (Figure 4E). As these metabolites represent the occurrence of metabolic changes complicated by depression during stroke recovery, further analysis of specific metabolites observed in PSD patients may help to distinguish PSD patients from controls and stroke patients (Supplementary Figure S2C).

Metabolic pathway enrichment analysis was performed using common genes, genes linked to excessive recovery, and specific metabolites. Valine, leucine, and isoleucine biosynthesis pathways were significantly enriched (Figure 4F). A detailed examination of the pathways associated with DEGs, the KEGG/metabolic-related pathways, and the three types of metabolites are listed above (Figure 3B, Supplementary Figure S2D). Additionally, we mapped significantly enriched disease-related metabolic pathways (Supplementary Figures S2E–H, Supplementary Figure S3 and Supplementary Figure S4).

We observed extensive interactions between the DEGs and metabolites (Figure 4G). Our analysis of the influence of differential metabolites and DEGs on the KEGG global metabolite network revealed that four metabolic pathways were significantly enriched, including the “metabolic pathway,” inositol phosphate metabolism, purine metabolism, and glycolysis/gluconeogenesis (Figure 4H). We were able to identify specific metabolites and DEGs that were extensively involved in long-term depression, the MAPK signaling pathway, the PI3K-Akt signaling pathway, and pathogenicity/*E. coli* infection in patients with PSD (Supplementary Figure S5). Our observations of differential metabolites in patients with PSD may reflect metabolic abnormalities which could be influenced by the intestinal flora.

### 3.5 Ecological landscape of gut microbes in patients with stroke and PSD

We performed α-diversity analysis to measure the diversity of the microbial communities within the samples. Reasonable amounts of sequencing data were acquired from the three groups, and species abundance was relatively high (Supplementary Figure S6). Therefore, we examined the biodiversity index of the sequencing volume of each sample at different sequencing depths, and we observed that the sequencing data volume of the three groups was large enough to reflect most microbial information in the samples (Supplementary Figure S6B). Our analysis also revealed high species abundance and species uniformity across the three groups (Supplementary Figure S6C). Additionally, we determined that this study possessed an appropriate sample size and high species richness (Supplementary Figure S6D).

**FIGURE 5:**
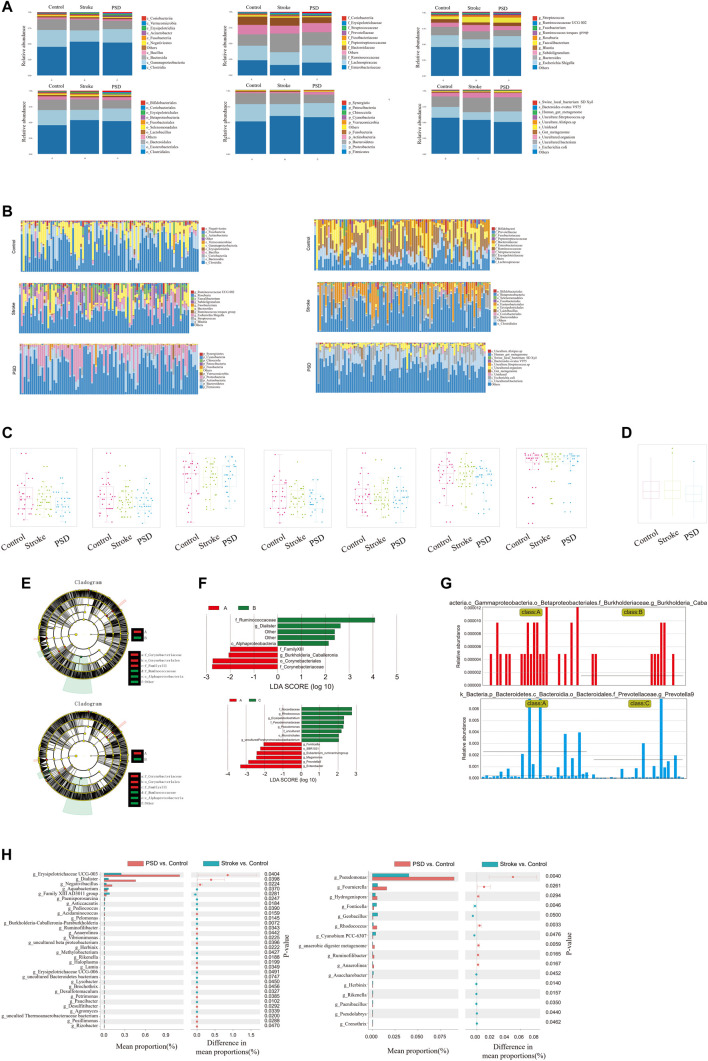
Ecological landscape of gut microbes in Control, stroke and PSD patients. **(A)**. Histogram of relative abundances of species in each group and classification level. **(B)**. Histogram of relative abundances of species in each grouping and classification level arranged as class, family, genus, order, phylum, and species from left to right and top to bottom. **(C)**. Box plots of differences in α diversity index between groups (from left to right: alpha diversity, ace index, chao1, good_coverage, observed_species, PD_whole_tree, Shannon, and Simpson index). **(D)**. Analysis of differences in β-diversity between groups based on weighted Unifrac distance. **(E)**. Evolutionary clade diagram. **(F)**. LDA core. **(G)**. Relative abundances of biomarkers in each group of samples. **(H)**. Results of STAMP analysis.

In regard to the species composition of gut microbes in the samples, we observed that the abundances of each group were different at each classification level (phylum, class, order, family, genus, and species; Figure 5A). The abundance of each sample was different at each taxonomic level (phylum, class, order, family, genus, and species; Figure 5B). Additionally, we observed that the abundance and diversity of intestinal flora were highest in controls, and this was followed by stroke patients and then PSD patients (Figure 5C).

To test for significant differences in the microbial communities among samples (groups), a β-diversity analysis was performed. Species diversity was highest in the samples collected from stroke patients, lower in controls, and lowest in PSD patients (Figure 5D). Based on the linear discriminant analysis effect size (LDASe) analysis, we determined that communities differing significantly between controls and stroke patients could be classified into six categories, while those differing between controls and PSD patients could be classified into five categories (Figure 5E). Based on the linear discriminant analysis, we identified the microbial population that played an important role in each group (Figure 5F). Species with an LDA value >2 were considered to be statistically significant biomarkers among the groups (Figure 5G).

Additionally, STAMP difference analysis was performed to identify species that differed significantly in terms of abundance among the groups. We observed that the abundance of g_Erysipelotrichaceae UCG-003 in the control samples was significantly different from that in the stroke patients, while g_*pseudomonas* abundance was significantly different between controls and patients with PSD (Figure 5H).

### 3.6 Intestinal microbes associate PSD with metabolic pathway disorders

**FIGURE 6:**
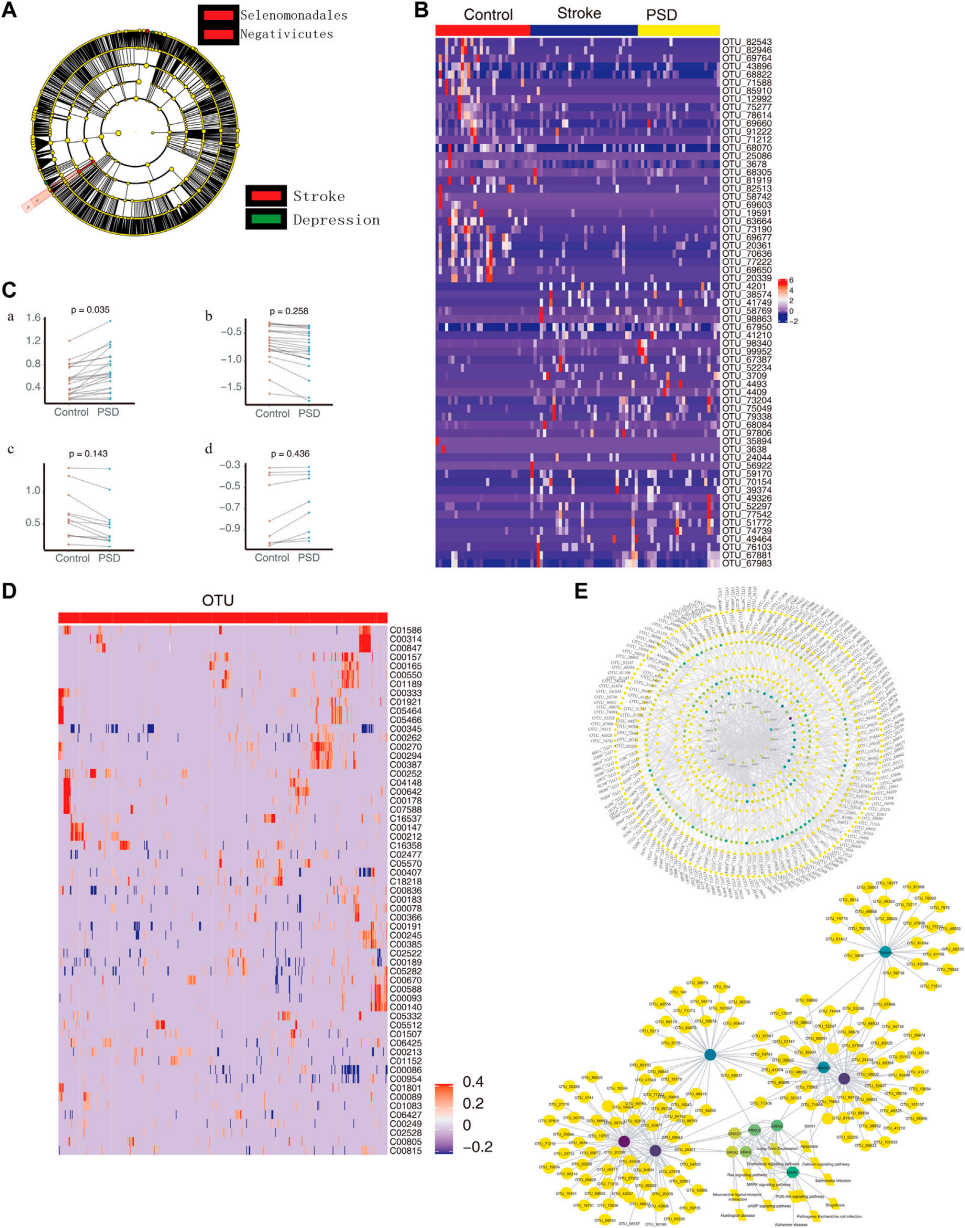
Intestinal microbes contribute to PSD depression with metabolic pathways. **(A)**. Evolutionary clade diagram. Group B indicates stroke patients; group C indicates PSD patients. **(B)**. Heatmap depicting the expression of common dysregulated microbes in healthy controls, stroke, and post-stroke depression patients. **(C)**. Paired scatterplot depicting changes in the abundance of common dysregulated microbes in stroke and post-stroke depression patients. **(D)**. Correlation between dysregulated microorganisms and dysregulated metabolites. **(E)**. Global control network depicting differentially expressed genes (hexagons), metabolites (circles), microorganisms (squares), and pathways (diamonds). Note: Group B consists of stroke patients, and group C consists of PSD patients.

When we compared the intestinal microbes between stroke patients and PSD patients using the LEfSe analysis, we observed that the communities exhibiting significant differences could be classified into two categories (Figure 6A). Based on LDA, we identified microbial groups that played an important role in each group of patients (Supplementary Figure S7A). Additionally, we identified biomarkers that were able to differentiate between the control and stroke patients or between the stroke and PSD patients (Supplementary Figure S7B). Based on the STAMP difference analysis, we determined that g_Erysipelotrichaceae UCG-014 abundance was significantly different between stroke and PSD patients (Supplementary Figure S7C).

Compared to the control group, we observed 65 common dysregulated intestinal microbes in patients with stroke and PSD (Figure 6B). During the development of stroke into PSD, 22 common dysregulated intestinal microbes underwent an initial upregulation (Figure 6Ca) that was followed by downregulation (Figure 6Cb). Of these, 12 common dysregulated microbes recovered after being upregulated and then returned to normal levels (Figure 6Cc). In contrast, nine other common dysregulated microbes recovered after downregulation but could not return to normal levels (Figure 6Cd). Furthermore, we identified a total of 474 depression-specific disordered microbes in PSD patients, and these microbes were able to distinguish among controls, stroke, and PSD patients (Supplementary Figure S7D). Additionally, a correlation analysis was performed to test the relationship between differential metabolites and differential microbes. There were 3,038 significant metabolite-microbe interactions, including 59 differential metabolites and 518 differential operational taxonomic units (Figure 6D). We also constructed a global regulatory network to explore the changes in microbes that affect phenotypic function (Figure 6E).

## 4 Discussion

The aim of this study was to gain a better understanding of the biological pathogenesis of PSD by examining the effects of intestinal flora, DEGs, and metabolites on the occurrence of depression in stroke patients. We determined that there were common DEGs in stroke and PSD patients, and we speculate that these DEGs may represent the basis of molecular disorders in PSD. Furthermore, various comorbidities, such as dementia, oxidative stress and obesity, and neuroinflammation have been frequently observed from stroke patients (Hermann et al., 2019; Popa-Wagner et al., 2020). It is reported that these syndromes highly related to altered metabolism (Chen et al., 2021; Noori et al., 2021; Riaz Rajoka et al., 2021; Chen et al., 2022). Delineating the correlation between intestinal flora and metabolism could help explain the role of intestinal flora in the development of the above comorbidities in stroke patients.

Our results indicate that intestinal flora could potentially mediate the dysregulation of gene expression in the cerebral cortex by altering metabolism in ways that increase the risk of depression. These findings provide new targets for the prevention and treatment of PSD.

The results of the enrichment analysis revealed that the DEGs were primarily involved in biological functions related to metabolism, immunity, microbes, and the nervous system. Consistent with these results, *Salmonella* infection was reported to be one of the causes of meningitis (Byer et al., 2017). Fatty acid metabolism and biosynthesis were determined to be closely related to neurological behavior and metabolic disorders (Augustin et al., 2018), and the Hippo signaling pathway has been reported to be essential for brain development (Ouyang et al., 2020). Additionally, the PI3K-Akt and NF-kb signaling pathways have been demonstrated to be involved in the occurrence of autism spectrum disorders, as they regulate inflammation-related biological functions (Zhang et al., 2020). These findings provide further support for our results that indicate that immune disorders and metabolic abnormalities are closely related to PSD.

Based on the findings of previous studies examining the disease-causing effects of metabolic abnormalities, we speculated that patients with PSD would experience changes in their metabolism. Indeed, we observed that these patients exhibited a high abundance of organic acids and derivatives, lipids and lipid-like molecules, and organic heterocyclic compounds. Previous studies have demonstrated that organic acids and their derivatives can regulate intestinal microflora, and this in turn affects the metabolism and immunity of the organism (Eyduran et al., 2015; Zhang et al., 2018). Lipids play an important role in regulating synaptic plasticity, brain metabolism, energy supply, and receptor phenotypes (Hussain et al., 2020), while heterocyclic compounds can improve brain function (Potkin et al., 2014). Therefore, we conducted further analyses to understand the effects of abnormal metabolites on brain function.

Tyrosine (Carvalho-Silva et al., 2019) and steroid metabolism (Qaiser et al., 2017) are closely related to brain function, while the tricarboxylic acid (TCA) cycle plays a key role in brain energy regulation and metabolism (Voss et al., 2020). Therefore, we speculated that abnormal metabolism may induce stroke. Our findings revealed that the purine metabolic pathway was significantly enriched in patients with stroke. Adenosine deaminase is an enzyme involved in purine metabolism and is important for the maintenance of the immune system and for the development and functioning of the central nervous system (Potkin et al., 2014). As normal purine metabolism is particularly important for normal brain function (Garcia-Esparcia et al., 2015), the observation in this study that purine metabolism is significantly enriched in stroke patients is of critical importance in regard to our understanding of the pathogenesis of depression.

Furthermore, we identified common abnormal metabolites in stroke and PSD patients, thus indicating that these metabolites could increase the risk of depression after stroke. In the present study, the levels of the N-methyl-d-aspirate receptor antagonist acamprosate were lower in stroke patients than they were in controls, and they were significantly higher in PSD patients compared to levels in controls. Acamprosate exerts a neuroprotective effect against brain damage caused by neural ischemia (Choi et al., 2019). Additionally, abnormal metabolites and DEGs in our analysis were observed to be significantly involved in pathways related to intestinal flora, metabolism, and the nervous system. The “metabolic pathway” purine metabolism, inositol phosphate metabolism, and glycolysis/gluconeogenesis pathway all exhibited significant enrichment. In the nervous system, different types of cells respond to external stimuli through the inositol lipid signaling pathway (Hanley et al., 1988), and glycolysis/gluconeogenesis is essential for maintaining normal energy metabolism. Studies have demonstrated that the glycolysis/gluconeogenesis pathway is closely related to Alzheimer’s disease (Foltynie, 2019). Therefore, our findings and the literature support the idea that intestinal flora can alter metabolism and thereby influence the risk of PSD.

Based on the 16S rDNA sequencing data, we determined that the abundance and diversity of intestinal flora were both higher in PSD patients than they were in controls and patients with stroke. This is an important finding, as the intestinal flora can influence the brain and the behavior of organisms through the brain-gut axis (Cryan et al., 2019). Additionally, correlation analysis revealed numerous connections between dysregulated metabolites and DEGs. Previous studies have demonstrated that inosine can repair damaged brain tissues (Levitsky et al., 2019), that guanosine has a neuroprotective effect on acute ischemic stroke (Connell et al., 2013), and that α-tocopherol can protect against brain damage and neuronal mitochondrial dysfunction (Murad et al., 2014). Adenine nucleoside has also been recognized as an endogenous neuroprotective agent that plays an important role in ischemic stroke (Levy et al., 2020). Furthermore, GNAO1 is closely related to epileptic encephalopathy and neurological dysfunction (Feng et al., 2017), PRKCB exerts a regulatory effect on neuronal function (Antonell et al., 2016), and GRIA2 is closely related to status epilepticus and depression (Gasparini et al., 2014). Therefore, we speculate that intestinal flora can contribute to depression in stroke patients by dysregulating gene expression in the cerebral cortex to thereby alter metabolism.

Our results must be considered in light of certain limitations. First, as the bioinformatics analyses were conducted based on retrospective data, our conclusions must to be validated using data from a prospective cohort. Second, different data sets were used for the transcriptomics analysis, 16S rDNA analysis, and non-targeted metabolomics, and this may increase heterogeneity and confounding. Finally, all patients in our study were obtained from a single site, and based on this, our results may not be applicable to different patient populations. Further studies must be conducted to gain a better understanding of the pathogenesis and treatment of PSD.

## Data Availability

The datasets presented in this study can be found in online repositories. The name of the repository and accession number can be found below: National Center for Biotechnology Information (NCBI) BioProject, https://www.ncbi.nlm.nih.gov/bioproject/, PRJNA838414.
