# Supplementary figures and images for: Intestinal flora induces depression by mediating the dysregulation of cerebral cortex gene expression and regulating the metabolism of stroke patients
Source: Front Mol Biosci. 2022 Nov 30;9:865788. doi: 10.3389/fmolb.2022.865788 (PMC9748625; doi:10.3389/fmolb.2022.865788)

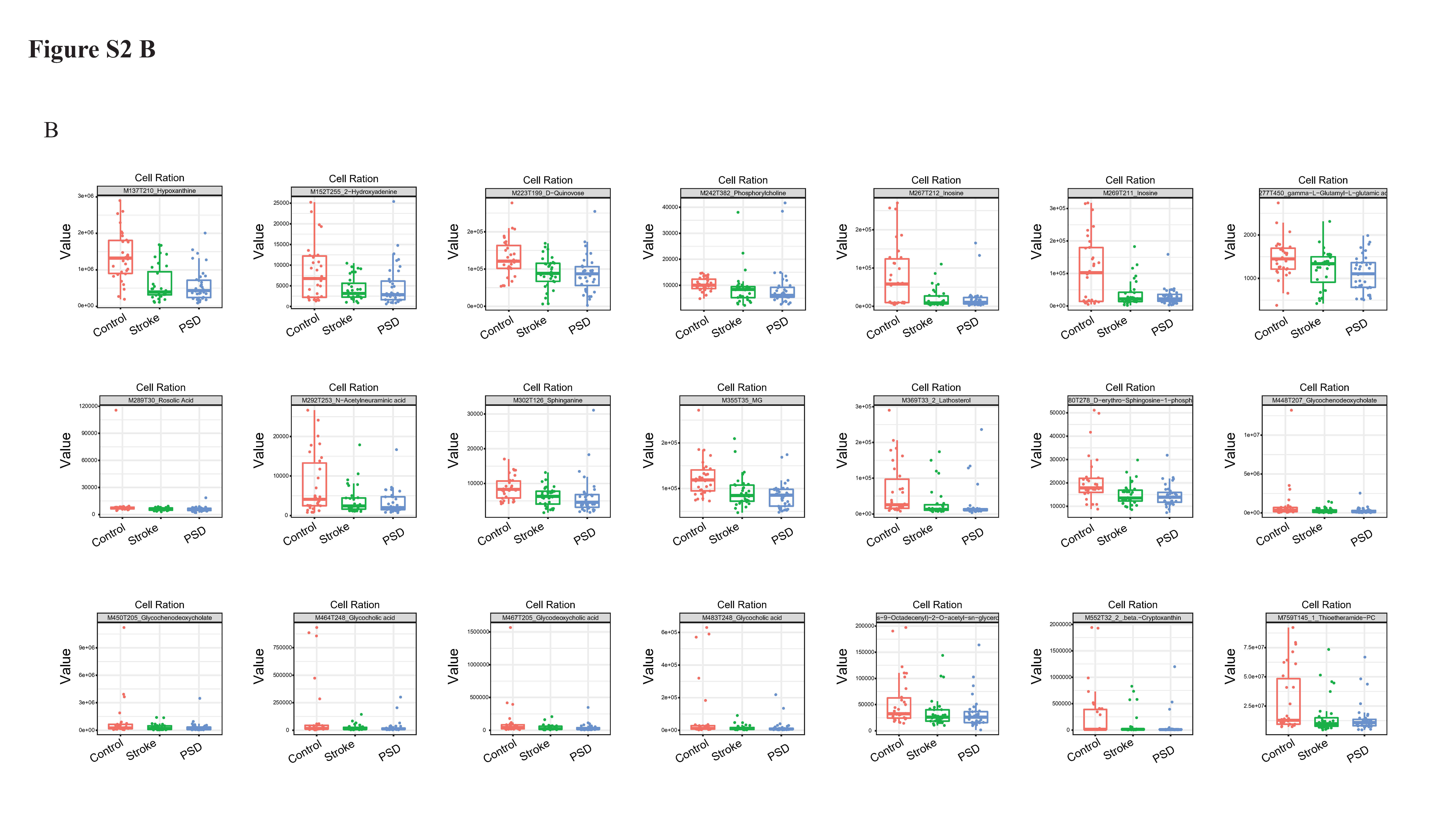

Supplement: Supplementary file 1 [file Image3.JPEG]

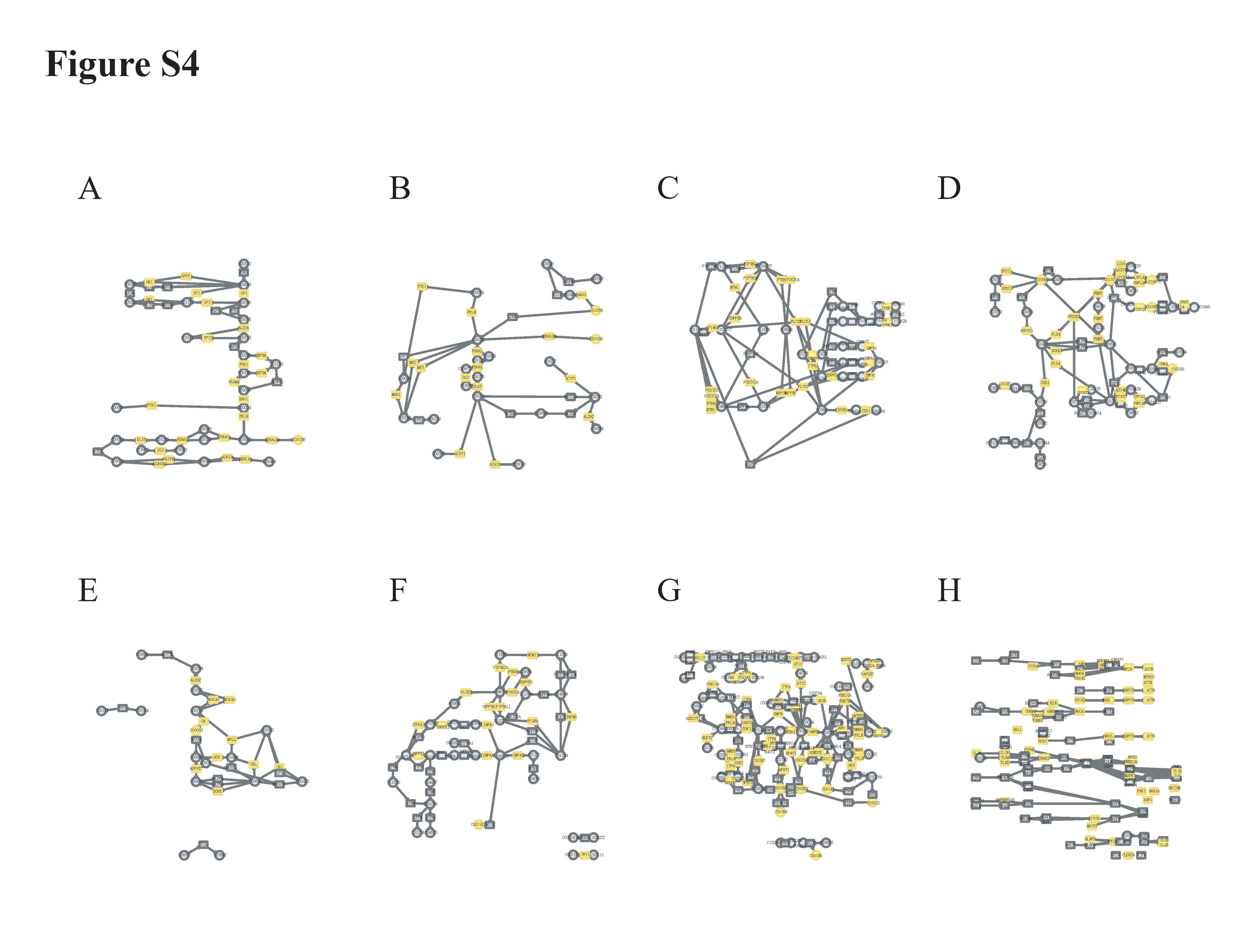

Supplement: Supplementary file 9 [file Image6.JPEG]
